# Caveolae in Rabbit Ventricular Myocytes: Distribution and Dynamic Diminution after Cell Isolation

**DOI:** 10.1016/j.bpj.2017.07.026

**Published:** 2017-09-05

**Authors:** Rebecca A.B. Burton, Eva A. Rog-Zielinska, Alexander D. Corbett, Rémi Peyronnet, Ilona Bodi, Martin Fink, Judith Sheldon, Andreas Hoenger, Sarah C. Calaghan, Gil Bub, Peter Kohl

**Affiliations:** 1 Department of Pharmacology, University of Oxford, Oxford, United Kingdom; 2 National Heart and Lung Institute, Imperial College London, London, United Kingdom; 3 Institute for Experimental Cardiovascular Medicine, University Heart Center Freiburg-Bad Krozingen, Faculty of Medicine, University of Freiburg, Freiburg, Germany; 4 Physics and Astronomy, University of Exeter, Exeter, United Kingdom; 5 Department of Physiology, Anatomy and Genetics, University of Oxford, Oxford, United Kingdom; 6 Department of Molecular, Cellular and Developmental Biology, University of Colorado, Boulder, Colorado; 7 School of Biomedical Sciences, University of Leeds, Leeds, United Kingdom

## Abstract

Caveolae are signal transduction centers, yet their subcellular distribution and preservation in cardiac myocytes after cell isolation are not well documented. Here, we quantify caveolae located within 100 nm of the outer cell surface membrane in rabbit single-ventricular cardiomyocytes over 8 h post-isolation and relate this to the presence of caveolae in intact tissue. Hearts from New Zealand white rabbits were either chemically fixed by coronary perfusion or enzymatically digested to isolate ventricular myocytes, which were subsequently fixed at 0, 3, and 8 h post-isolation. In live cells, the patch-clamp technique was used to measure whole-cell plasma membrane capacitance, and in fixed cells, caveolae were quantified by transmission electron microscopy. Changes in cell-surface topology were assessed using scanning electron microscopy. In fixed ventricular myocardium, dual-axis electron tomography was used for three-dimensional reconstruction and analysis of caveolae in situ. The presence and distribution of surface-sarcolemmal caveolae in freshly isolated cells matches that of intact myocardium. With time, the number of surface-sarcolemmal caveolae decreases in isolated cardiomyocytes. This is associated with a gradual increase in whole-cell membrane capacitance. Concurrently, there is a significant increase in area, diameter, and circularity of sub-sarcolemmal mitochondria, indicative of swelling. In addition, electron tomography data from intact heart illustrate the regular presence of caveolae not only at the surface sarcolemma, but also on transverse-tubular membranes in ventricular myocardium. Thus, caveolae are dynamic structures, present both at surface-sarcolemmal and transverse-tubular membranes. After cell isolation, the number of surface-sarcolemmal caveolae decreases significantly within a time frame relevant for single-cell research. The concurrent increase in cell capacitance suggests that membrane incorporation of surface-sarcolemmal caveolae underlies this, but internalization and/or micro-vesicle loss to the extracellular space may also contribute. Given that much of the research into cardiac caveolae-dependent signaling utilizes isolated cells, and since caveolae-dependent pathways matter for a wide range of other study targets, analysis of isolated cell data should take the time post-isolation into account.

## Introduction

Caveolae (Latin for “little caves”), cholesterol- and sphingolipid-rich spheroid plasma membrane domains of 50–100 nm diameter, are found in close proximity to the surface sarcolemma of cells, usually linked to it via a “bottle-neck”-like connection (1, 2). Caveolae are present in many cell types, including those of the cardiovascular system (cardiomyocytes, endothelial cells, fibroblasts, smooth muscle cells) (3). A defining feature of caveolae is the presence of specialized scaffolding proteins—caveolins and cavins (4, 5, 6). Caveolins (Cav-1–Cav-3) are responsible in part for the spheroid morphology of caveolae, through their asymmetrical membrane insertion and their tendency to form oligomers that promote local concave membrane invagination (7). Cav-3 is a muscle-specific isoform (8, 9), whereas Cav-1 is widely expressed in many cell types, including adipocytes, endothelial cells, pneumocytes, and fibroblasts (9, 10, 11). The muscle-specific Cav-3 is essential for caveolae formation in cardiomyocytes, and Cav-3-deficient mice develop cardiomyopathies (12, 13, 14, 15, 16).

Since their discovery in the 1950s, caveolae have been shown to play essential roles in a wide range of cellular processes, including signal transduction (17, 18, 19), macromolecular complex trafficking (20, 21), and—owing to the presence of several ion-channel and exchanger proteins in caveolar membranes—electrophysiology (22, 23, 24, 25, 26). It is thought that caveolae thus segregate and integrate certain signaling pathways in microdomains of the plasma membrane.

Their shape and composition enable surface sarcolemmal caveolae in skeletal (27) and cardiac muscle (28) to act as “spare” plasma membrane, which can be recruited during mechanical perturbations, such as stretch. Indeed, mechanical stretch or osmotic swelling can lead to sarcolemmal membrane incorporation of surface-sarcolemmal caveolae, preventing excessive sarcolemmal membrane tension (27, 28, 29, 30, 31, 32, 33). The dynamic recruitment of surface-sarcolemmal caveolae into the surface membrane increases membrane capacitance (29) and affects the density and distribution of sarcolemmal ion channels (18). Depletion of surface-sarcolemmal caveolae in turn prevents the stretch-induced increase in membrane capacitance and inhibits the slowing of conduction otherwise seen upon mechanical distension of the intact heart (29). Surface-sarcolemmal caveolae are therefore one of the mechano-sensors/-transducers of cardiomyocytes (23, 28, 34, 35).

In cardiac and skeletal muscle, Cav-3 is distributed throughout the sarcolemma, including external surface and transverse-tubular (T-tub) membranes, as visualized by immunofluorescence (24, 36). Nonetheless, caveolae are traditionally thought to be structures associated with the “outermost” surface sarcolemma only, despite reports (37, 38, 39) suggesting the presence of caveolae in T-tub membranes. However, the presence and relevance of T-tub caveolae have remained controversial (37, 40, 41). Here, we describe in detail the three-dimensional (3D) organization of caveolae at surface sarcolemmal and T-tub membranes in rabbit ventricular myocardium.

The rabbit model was chosen because it represents a species that mimics human cardiac physiology surprisingly well. The characteristics of the rabbit heart resemble those of the human heart in terms of structure (42, 43), regional contractile and diastolic properties (44), and responses to pathophysiological stimuli (such as ischemia (43, 45)). Key electrical features (such as action potential properties) and responses to relevant pharmacological interventions also show pronounced similarities between the two species (46, 47, 48). Additionally, rabbit cardiomyocyte ultrastructure (for example, of the T-tub system) is closely reminiscent of that in human cells (49). As a consequence, there is a growing appreciation for the utility of rabbit models to study basic electrophysiological concepts, human heritable diseases (50), and for preclinical cardiac safety testing (51).

Isolated ventricular cardiomyocytes are an important model system in molecular and cellular cardiology, and they are used in many studies of caveolar signaling. At the same time, the extent to which structural and functional in vivo properties of caveolae are preserved in vitro is unknown. What is known is that isolated myocytes, even if optimally isolated and maintained, gradually change shape, structure, and function post-isolation, towards a less in-vivo-like state (52). This includes a progressive reduction in T-tub density, by ∼40% within 24 h in rabbit (53, 54) and by ∼60–100% within 72 h in rat (55). These structural changes have marked effects on cell physiology, including excitation-contraction coupling. Here, we document a dynamic reduction in the presence of near-surface caveolae in rabbit ventricular cardiomyocytes within the first 8 h post-isolation—a time course that is directly relevant for research on acutely isolated cells.

## Materials and Methods

All investigations conformed to the United Kingdom Home Office guidance on the Operation of Animals (Scientific Procedures) Act of 1986.

### Heart and cell isolation

New Zealand white rabbit hearts (*n* = 6 for intact tissue studies) were swiftly excised after Schedule 1 killing, Langendorff perfused with normal Krebs-Henseleit solution (containing 118 mM NaCl, 4.75 mM KCl, 2.5 mM CaCl_2_, 24.8 mM NaHCO_3_, 1.2 mM MgSO_4_, 1.2 mM KH_2_PO_4_, 11 mM glucose, and 10 U/L insulin (pH 7.4)) and then cardioplegically arrested using high K^+^ (25 mM). For tissue fixation, cardioplegically arrested hearts were fixed by coronary perfusion with iso-osmotic Karnovsky’s reagent (56) (2.4% sodium cacodylate, 0.75% paraformaldehyde, and 0.75% glutaraldehyde).

For cell isolation, hearts isolated as above (*n* = 3 for imaging, *n* = 10 for patch-clamp recordings) were Langendorff perfused with normal Tyrode solution (containing 140 mM NaCl, 5.4 mM KCl, 5 mM HEPES, 1.8 mM CaCl_2_, 1 mM MgCl_2_, and 11 mM glucose (pH 7.4)). The myocardium was enzymatically digested with collagenase and ventricular cardiomyocytes were isolated. After isolation, cells were held in a storage solution (containing 140 mM NaCl, 5.4 mM KCl, 1 mM MgCl_2_, 5 mM HEPES, 11 mM glucose, 1.8 mM CaCl_2_, 0.17 g/L trypsin inhibitor, and 1 g/L bovine serum albumin (pH adjusted to 7.4 with NaOH)) at room temperature until recording/fixation. This solution was refreshed every 2 h.

All solutions were controlled for iso-osmolality (295–305 mOsm).

### Electron tomography of ventricular myocardium

Fixed tissue was washed with 0.1 M sodium cacodylate, post-fixed in 1% OsO_4_ for 1 h, dehydrated in graded acetone, and embedded in Epon-Araldite resin. Thick sections (275 nm) were cut, transferred onto copper slot grids, and post-stained with 2% aqueous aranyl acetate, followed by Reynolds’ lead citrate. Colloidal gold particles (15 nm) were added to both surfaces of the sections to serve as fiducial markers for post-processing tilt series alignment.

Preparations were imaged at the Boulder Laboratory for 3D Electron Microscopy of Cells (University of Colorado at Boulder, Boulder, CO) using an intermediate-voltage electron microscope (Tecnai F30; FEI-Company, Eindhoven, the Netherlands) operating at 300 kV, with images captured on a charge-coupled device camera (UltraScan; Gatan, Pleasanton, CA), at a pixel size of (1.206 nm)^2^. For dual-axis tomography, a series of tilted views was collected from +60° to −60° at 1° increments. After the first tilt series was acquired, the specimen was rotated by 90° in the horizontal plane and another +60° to −60° tilt series was taken. The images from each tilt series were aligned by fiducial marker tracking and back projected to generate two single full-thickness reconstructed volumes (tomograms), which were then combined to generate a high-resolution 3D reconstruction of the original partial cell volume (57, 58, 59). All tomograms were processed and analyzed using the IMOD software, which was also used to generate 3D models of the relevant structures of interest (60). Models were smoothed and meshed to obtain the final 3D representation, in which spatial relationships of surface sarcolemma, T-tub, and caveolae were visualized.

### Transmission electron microscopy of isolated cardiomyocytes

Cells were fixed (at 0, 3, and 8 h post-isolation) using iso-osmotic Karnovsky’s reagent (57), embedded in LR white (Agar Scientific, Stansted, United Kingdom), and sectioned at 80 nm thickness (Reichert-Jung Ultracut; Ametek Reichert Technologies, Depew, NY). Sections were post-stained with 2% aqueous uranyl acetate, followed by Reynolds’ lead citrate. Cells were imaged by transmission electron microscopy (TEM) (1200EX II; JEOL, Tokyo, Japan).

### Scanning electron microscopy of isolated cardiomyocytes

Cells were fixed as above, treated with 1% osmium tetroxide for 1 h, washed in phosphate-buffered saline, dehydrated in ethanol, and subjected to HMDS (hexamethyldisilazane) treatment. The protocol involved the following steps: transfer of cells on to a coverslip, ethanol dehydration series, drying for 3 min with HMDS, transfer onto a scanning electron microscopy (SEM) stub, coating with gold in a sputter coater, and imaging using SEM (JEOL JSM-5510; for details, see (61)).

For analysis of changes in area, diameter, and circularity (ratio of short to long axis) of sub-sarcolemmal mitochondria, SEM images were analyzed using custom-written software (available by request from G.B.). Custom software (written in the Java programming language) allowed the operator to outline pixels containing mitochondria. The sub-image within the user-generated outline was auto-scaled and thresholded to select pixels corresponding to the mitochondria, and dilation operations were applied to the pixels to obtain a contiguous (filled) region that corresponded to the mitochondrion’s shape. The area of each mitochondrion is calculated by summing pixels in the contiguous region. The operator then uses an on-screen ruler to find the long and short axis of the mitochondria, and circularity is calculated from their ratio. The software generates an intensity profile for the ruler’s path by summing pixel intensity values for three pixels perpendicularly on either side of the path along its entire length. Peaks in the intensity profile are used to measure distances between features in the image (62).

Cells were randomly distributed relative to the observation angle of SEM image acquisition. Although this increases the standard deviation of measurements (due to the cosine error), it does so in equal measure for all time points.

### Membrane capacitance measurements

The patch-clamp technique (Axopatch 200B; Axon Instruments, Union City, CA) was used to record plasma membrane capacitance in whole-cell mode. Cells were allowed to rest after isolation for 2 h. To match structural observations, patch-clamp data were binned into 0–3, 4–5, and 6–8 h post-isolation (although the first group effectively contains data collected during the third hour only; it is accordingly a smaller sample). Data were analyzed using pClamp10 from a standard pulse protocol (5 mV amplitude, 10 ms duration) to determine the value of cell capacitance. Capacitance was recorded immediately after membrane rupture; recordings with access resistance over 10 M*Ω* were rejected.

### Statistics

All values are expressed as the mean ± SE, and statistical significance was assessed by one-way analysis of variance.

## Results

**Figure 1:**
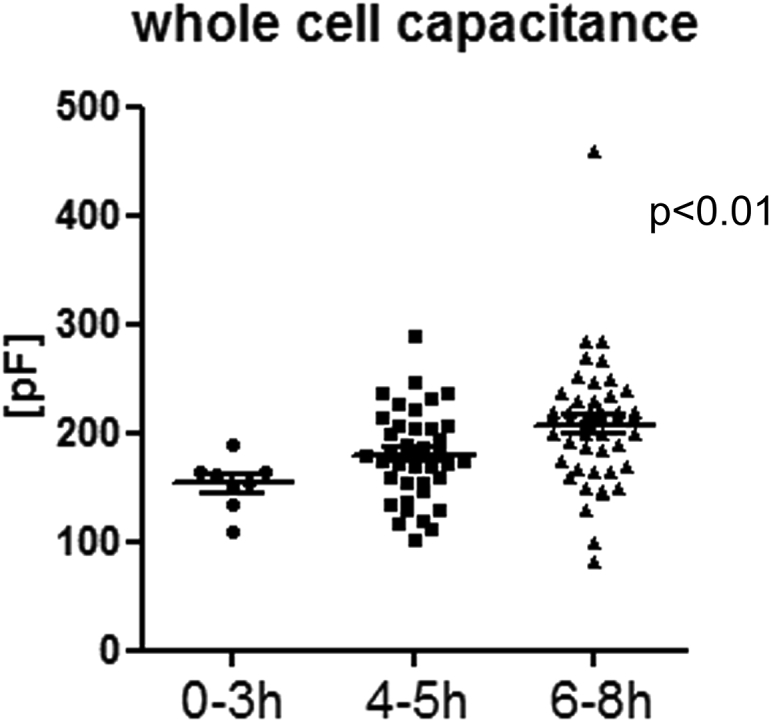
Whole-cell plasma membrane capacitance recordings of rabbit ventricular myocytes, showing a gradual increase over time post-isolation. Data were analyzed using one-way analysis of variance, with significance indicated for the overall effect of time; *n* = 8 (0–3 h), *n* = 40 (4–5 h), *n* = 48 (6–8 h) cells.

Dynamic changes in cardiomyocyte plasma membrane surface area were monitored by whole-cell membrane capacitance measurement (63) using the patch-clamp technique. Cell capacitance gradually increased over an 8 h period post-isolation (Fig. 1).

### Surface-sarcolemmal caveolae numbers in ventricular cardiomyocytes post-isolation

**Figure 2:**
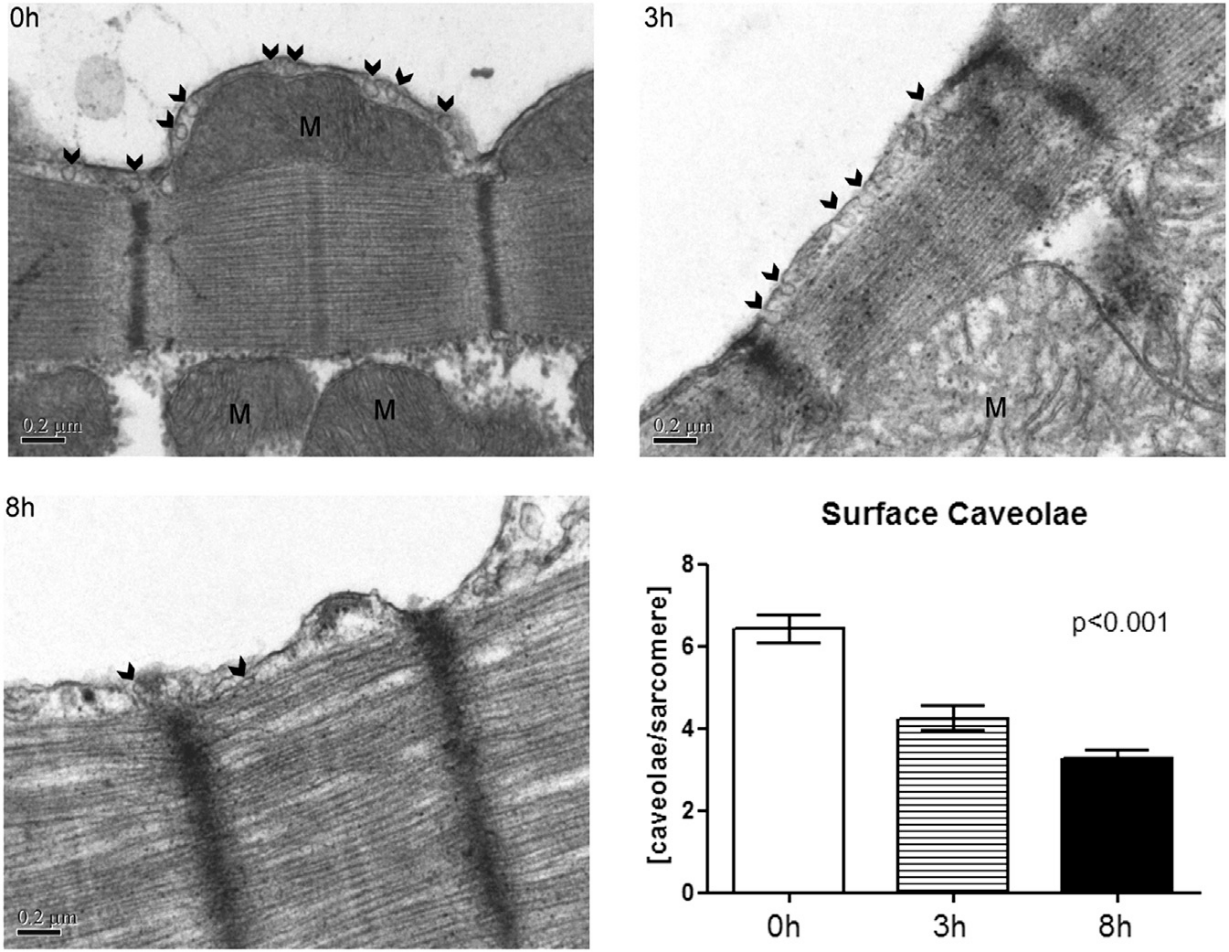
TEM images showing changes in the distribution of caveolae (surface-sarcolemmal caveolae, examples indicated by *arrows*) in rabbit ventricular myocytes at 0, 3, and 8 h post-isolation. The number of surface-sarcolemmal caveolae is scaled per membrane spanning two neighboring *Z*-lines (“per sarcomere”). This number declined with time after cell isolation. Surface-sarcolemmal caveolae were quantified for 9–12 sarcomeres per cell, in six myocytes at each time point. Data were analyzed using one-way analysis of variance, with significance indicated for overall effect of time. M, mitochondria. Scale bars represent 0.2 *μ*m.

The ultrastructure of the surface membrane and the distribution of surface-sarcolemmal caveolae were studied over time after isolation using TEM. Surface-sarcolemmal caveolae were defined as either visibly connected to the surface sarcolemma or separate from the plasma membrane by ≤100 nm (measured from the surface-sarcolemmal caveola center) in a given TEM image. There was a significant decrease over time post-isolation (by 34% at 3 h and by 49% at 8 h post-isolation, when compared to 0 h) in the overall number of surface-sarcolemmal caveolae per membrane segment spanning two neighboring *Z*-lines (henceforth referred to as “per sarcomere”; Fig. 2; Table 1). Additionally, we observed an overall reduction in total cellular Cav-3 protein after 8 h post-isolation (Fig. S1).

**Table 1:** Surface-Sarcolemmal Caveolae: Presence in Intact Tissue and in Isolated Cardiomyocytes over Time Post-isolation

Time Point	Surface-Sarcolemmal Caveolae/Sarcomere (Mean ± SE)	Sarcomere/Cells Analyzed	Fractional Change (versus Intact Tissue)
Reference: intact tissue (ET)	6.22 ± 0.64	26 sarcomeres/21 cells	1.00
0 h post-isolation	6.41 ± 0.35	54 sarcomeres/6 cells	1.03
3 h post-isolation	4.24 ± 0.31	59 sarcomeres/6 cells	0.68 (0.66 vs. 0 h)
8 h post-isolation	3.27 ± 0.19	60 sarcomeres/6 cells	0.53 (0.51 vs. 0 h)

Data for intact tissue are from ET and those for isolated cardiomyocytes are from TEM. Surface-sarcolemmal caveolae/sarcomere in ET data includes an 80-nm-wide sarcolemmal strip, connecting two *Z*-lines, mimicking the section thickness of TEM.

### Changes in surface topology of ventricular cardiomyocytes post-isolation

**Figure 3:**
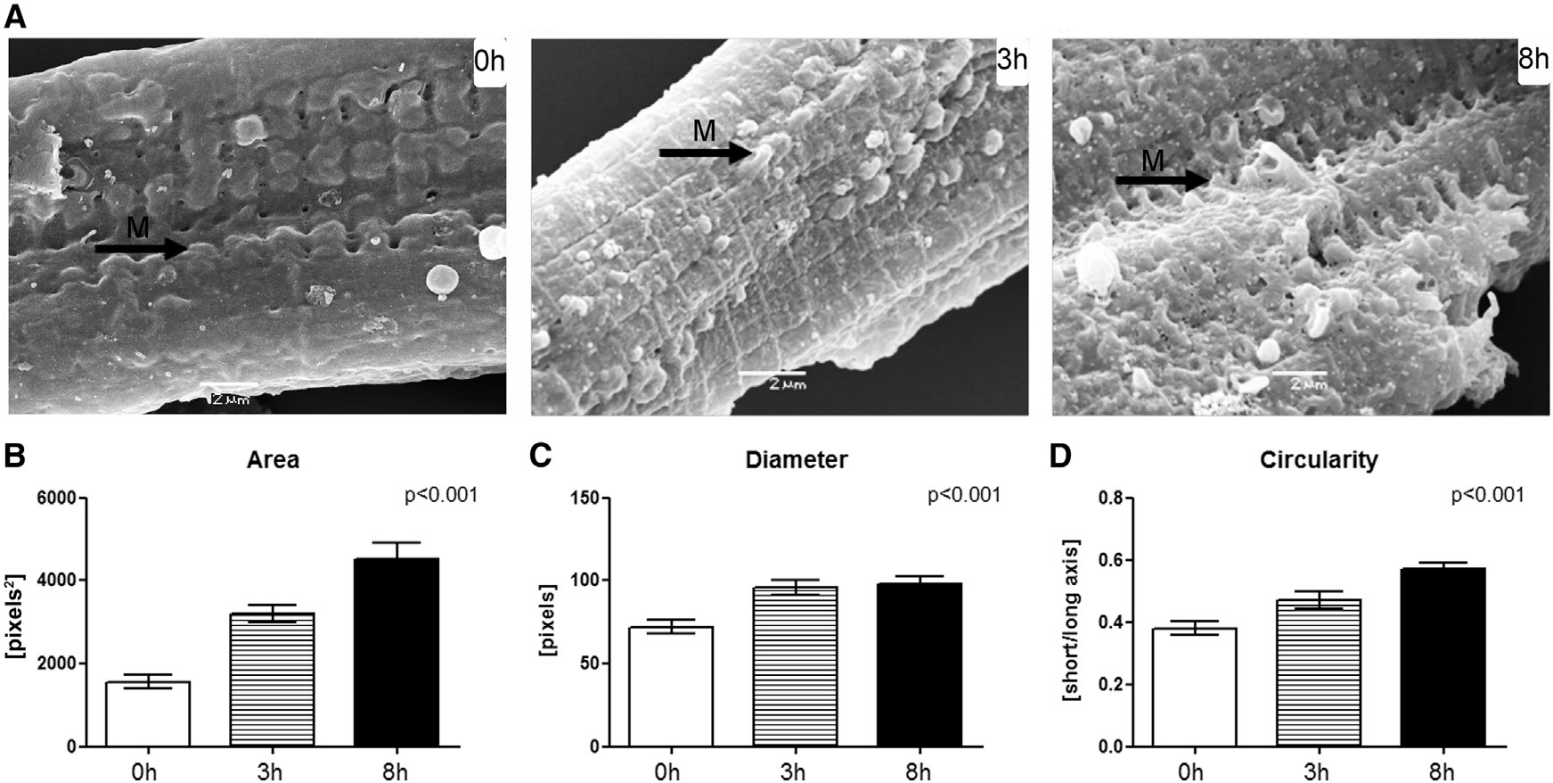
(*A*) SEM images of cardiomyocyte surface topology at 0, 3, and 8 h post-isolation, with protrusions of the sarcolemmal membrane (*arrows labeled M*) increasingly prominent. (*B*–*D*) Area (*B*), diameter (*C*), and circularity (*D*) of membrane protrusions increased with time (3 cells per time point; mitochondria *n* = 40 (0 h), *n* = 27 (3 h), and *n* = 39 (8 h)). Significance is indicated for the overall effect of time. Scale bars represent 2 *μ*m.

SEM revealed an increase in area, diameter, and circularity of sub-sarcolemmal membrane protrusions, commonly associated with sites of mitochondria (Fig. 3). Protrusions typically correspond to a single mitochondrion (e.g., Fig. 2, 0 h). Observed changes in size and shape are indicative of mitochondrial swelling.

### Intracellular caveolae distribution in ventricular tissue and isolated cells

**Figure 4:**
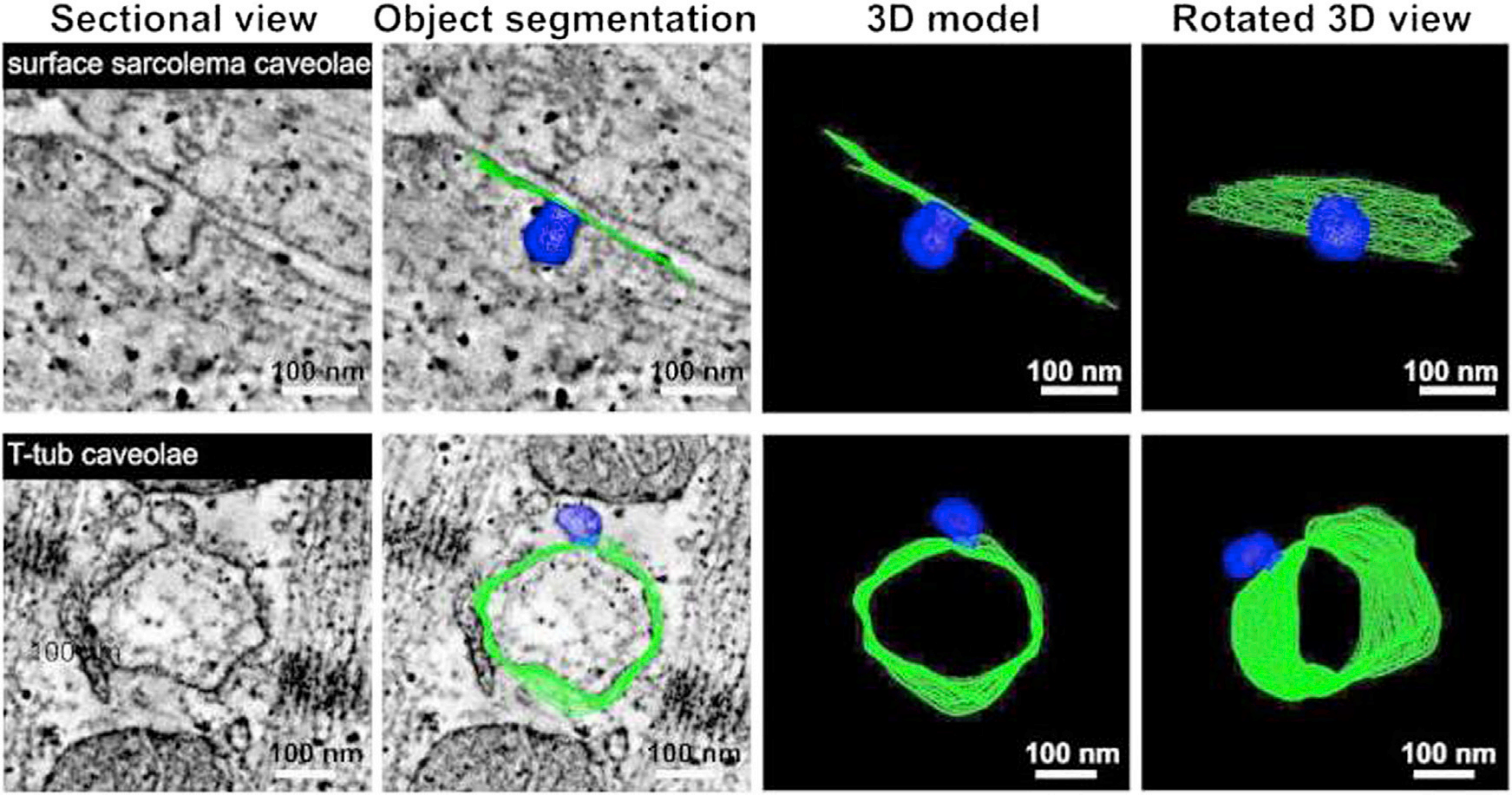
Representative ET images of surface-sarcolemmal caveolae (*top row*) and T-tub caveolae (*bottom row*) in rabbit ventricular tissue. Dual-axis ET was used to image, reconstruct, and model surface-sarcolemmal and T-tub caveolae and their host membranes in 3D. Isotropic voxel size was (1.206 nm)^3^ and *z*-depth was 275 nm; host membrane surfaces are indicated in green and caveolae in blue.

Using electron tomography (ET), we quantified the presence of caveolae in intact left ventricular rabbit tissue (Fig. 4; Movie S1). Surface-sarcolemmal caveolae were found at a frequency of 6.22 ± 0.64 caveolae/sarcomere (per 80-nm-wide strip connecting two Z-lines), similar to the value seen in freshly isolated cardiomyocytes (Table 1).

Additionally, we observed the regular presence of T-tub caveolae in intact myocardial tissue (Fig. 4; Movie S2). T-tub caveolae were seen in 133 (63%) of 209 T-tub segments analyzed (average segment length: 275 nm) and in every cell tested. The density of T-tub caveolae was lower than that of surface-sarcolemmal caveolae (3.23 ± 0.3 vs. 12.32 ± 1.26, respectively, per 1 *μ*m^2^ membrane area). These structures were different from the T-tub folds described previously in murine myocytes (64).

There was no significant difference between surface area, volume, and major axis diameter of caveolae from the different sub-cellular locations (Table 2).

**Table 2:** Surface-Sarcolemmal and T-Tub Caveolar Dimensions in Intact Ventricular Tissue

Caveolae Type	Longest Axis Length (nm)	Volume (nm^3^)	Surface (nm^2^)
Surface-sarcolemmal caveolae (n = 45)	77.4 ± 2.42	(276 ± 19) × 10^3^	(20.32 ± 0.02) × 10^3^
T-tub caveolae (*n* = 64)	73.9 ± 2.38 (n.s.)	(244 ± 19) × 10^3^ (n.s.)	(19.75 ± 0.01) × 10^3^ (n.s.)

Data are from reconstructions. n.s., no significant difference between surface-sarcolemmal and T-tub caveolae.

**Figure 5:**
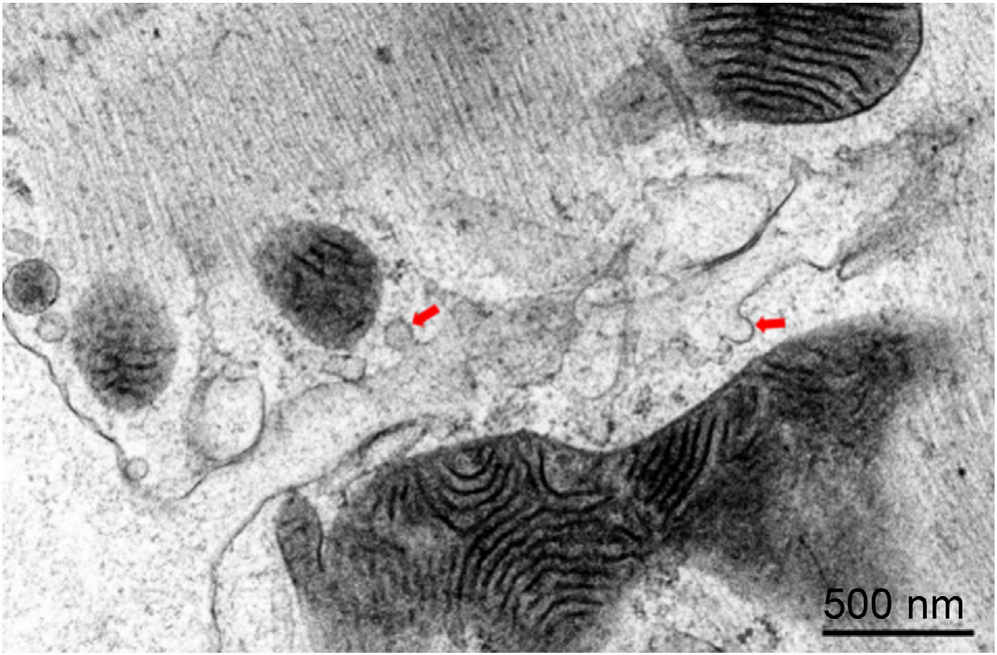
T-tub-caveola-compatible structures (*arrows*) identified in 2D (thin section) TEM images of freshly isolated (0 h) rabbit ventricular cardiomyocytes. Scale bars represent 500 nm.

**Figure 6:**
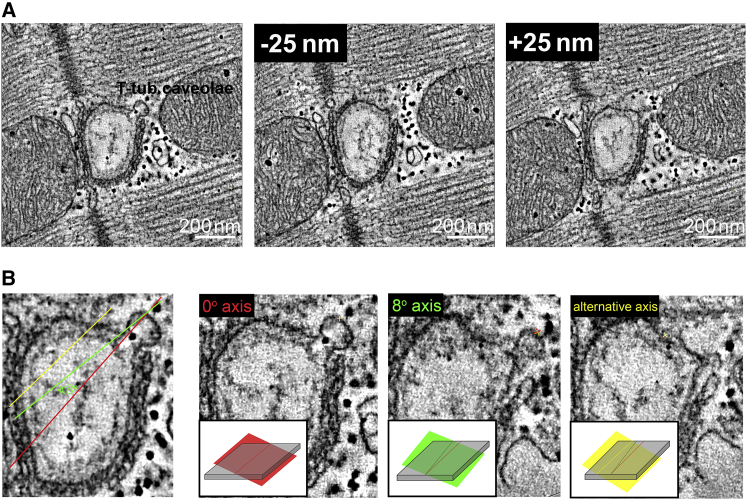
Illustration of technical challenges associated with identifying T-tub caveolae in 2D sections. A tomographic slice containing a representative T-tub caveola is virtually sectioned along different planes to simulate TEM-like data with arbitrary sectioning planes relative to structures of interest. Both the level (*A*) and the angle (*B*) of the cutting plane (*shaded area* illustrating the thickness of a typical TEM slice) relative to the imaged structure affect the likelihood of positively identifying T-tub caveolae in non-3D data sets. Scale bars represent 200 nm.

With our knowledge of the presence of T-tub caveolae in rabbit ventricular cardiomyocytes, re-examination of TEM images revealed T-tub caveolae-like structures in 2D micrographs of isolated cells (Fig. 5). However, given the small radius of T-tub membrane curvature compared to the sarcolemmal surface, the nature and interrelation of probable T-tub caveolae with the T-tub surface membrane cannot be established with certainty in 2D sections (see Fig. 6).

### Presence of surface-sarcolemmal and T-tub caveolae in TEM images: spatial density considerations

The relatively abundant presence of T-tub caveolae in the 3D EM data runs counter to general perception, based on decades of experience with TEM. How might this come about?

Based on experiments with cell capacitance recordings before and after de-tubulation, one can conclude that between one-third and two-thirds of the total cell surface membrane is contained in T-tub (53). If, for ball-park estimation, one assumes a 50:50 split, and considers the 1:4 difference in T-tub caveolae density per unit of membrane compared to surface-sarcolemmal caveolae, there should be four times more surface-sarcolemmal caveolae in a single cell than T-tub caveolae. This difference alone does not explain the hitherto rare detection of T-tub caveolae in ventricular myocyte TEM work.

**Figure 7:**
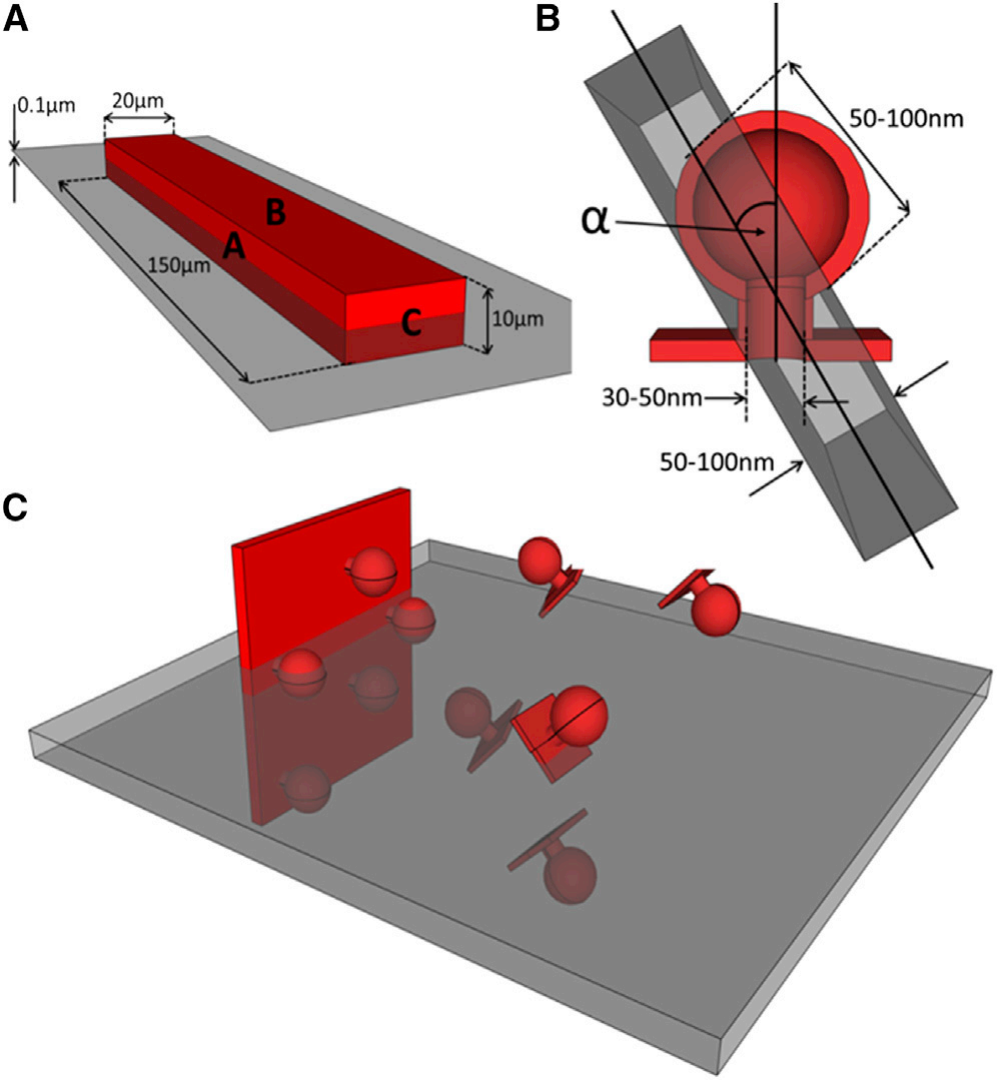
(*A*) Model cardiomyocyte (*red*) showing approximate interrelations of surfaces A–C with a TEM cutting plane (*gray*). (*B*) Cutaway through a model caveola showing approximate dimensions. The TEM section is oriented at an angle *α* relative to the caveola-centric orientation that would be optimal for identification. (*C*) Illustration showing the different likelihood of imaging caveolae on the sarcolemma (*left*) at an angle amenable for identification compared to that of imaging randomly oriented caveolae on a T-tub.

Fig. 7 *A* illustrates the spatial relations between a typical TEM section (thickness ∼0.1 *μ*m) and a cardiac myocyte (approximate dimensions H × W × L = 10 × 20 × 150 *μ*m). The total cell surface is made up of 2A + 2B + 2C. Given the high aspect ratio of the cell, areas A/B/C are proportional to 15:30:2, so that for the most part, the contribution from C can be ignored. Therefore, considering a TEM section through the long axis of the cell (as illustrated in Fig. 7 *A*), the relevant contributions to the presence of surface-sarcolemmal caveolae will come from intersections of the TEM sample with the two A-surfaces. These surfaces will contain a fraction of the total surface-sarcolemmal caveolae, equivalent to 2A/(2A + 2B), or—using the above ratio of surface areas—1/3 of all surface-sarcolemmal caveolae in that cell. Any 0.1 *μ*m TEM section cutting through this 10-*μ*m-high cell will therefore contain 0.01 × 1/3 of all surface-sarcolemmal caveolae. By comparison, as the sections will cut through the entire volume of the cell, a single section will contain 0.01 of all T-tub caveolae. Combining this with the relative host-surface densities (four times higher density for surface-sarcolemmal caveolae than T-tub caveolae), the difference in the numbers of surface-sarcolemmal caveolae and T-tub caveolae contained in any given TEM section is small (surface-sarcolemmal caveolae__TEM_ = (4/3) × T-tub caveolae__TEM_). This makes it even more surprising that T-tub caveolae have not been reported more regularly in previous TEM studies.

### Identifiability of surface-sarcolemmal and T-tub caveolae in TEM images

If there is a similar net presence of surface-sarcolemmal and T-tub caveolae per whole-cell TEM section, the question arises as to why T-tub caveolae may still end up being under-detected. To identify caveolae beyond doubt, a TEM section should run through the lumen of the “bottle-neck” connecting the circular caveola structure to the host membrane. In Fig. 7 *B*, the caveola-centric line, running parallel to the axis of the neck, illustrates the orientation of an ideally angled and positioned TEM section. Shown in gray and at an angle *α* to this line is the case for a TEM sectioning plane that runs through the lumen of the caveola connection at a non-ideal angle. If *α* exceeds a critical level (*α*_C_), the presence of a caveolar structure will not be confirmed even if the cut runs through the center of the neck area. If, in addition, the TEM sectioning plane is offset laterally (not running through the neck), one would see a caveola-sized circular structure whose connection to the host membrane cannot be confirmed, so one would not be able to positively identify this, for example, as T-tub caveolae, based on 2D TEM imaging data alone (see Fig. 6 *B*).

The likelihood that any TEM section plane will run through the neck region of randomly distributed surface-sarcolemmal and T-tub caveolae may be assumed to be identical. However, the likelihood of a favorable angular orientation of surface-sarcolemmal or T-tub caveolae with TEM sectioning differs. This is illustrated in Fig. 7 *C*. While surface-sarcolemmal caveolae will generally be orientated roughly orthogonal to the outer cell surface, T-tub caveolae may be aligned in any direction, depending on the orientation of the host T-tub (which, in rabbit, includes spoke-like transversal and additional cell-axis parallel segments (65)). From these, T-tub caveolae can protrude radially in all surface-orthogonal directions. The question then is what fraction of caveolae, cut at an optimal level—i.e., through the center of the neck region—will be positively identifiable as caveolae in TEM images.

The angle, *α*_C_, defines a cone that contains all TEM imaging planes that will yield an identifiable image of caveolae. The volume fraction of this cone, relative to a half-sphere, equals 1 − cos *α*_C_. An estimate of *α*_C_ has been obtained by simulating TEM sections in the 3D ET data, with estimates for the value of *α*_C_ varying between 8° and 20° and the average value being 13° (see example in Fig. 6 *B*). This correlates to a fraction of possible TEM sectioning planes relative to caveolar orientation that permit positive identification of ∼2.5% (at *α*_C_ =13°; ranging from 1.0% at 8° to 6.0% at 20°).

If one assumes, therefore, that T-tub caveolae can be randomly distributed and oriented in the cell (Fig. 7 *C*), one should expect to be able to identify no more than a few percent of those that are present in a TEM section *and* cut favorably (i.e., through the lumen of the neck region). In contrast, for surface-sarcolemmal caveolae, identifiability—if cut through the neck-region—will be close to 100% for two reasons. First, they are aligned nearly perpendicular to the cell surface membrane. Second, TEM imaging of densely populated cardiac myocytes is user biased toward alignment of sectioning planes with either the long or the short axis of the cell’s contractile lattice structures—and hence more or less perpendicular to the sarcolemma—the host membrane of surface-sarcolemmal caveolae.

As an aside, reliable identification of caveolae by definition also includes recognition of the host membrane, which is usually much more straightforward for surface sarcolemma, compared to less regular and variably orientated intracellular membrane systems. If one further takes into account the “best-case scenario” of histologically convincing identification, i.e., a cut that not only captures T-tub caveolae favorably (right level and orientation) but also runs either longitudinally through a suitably long section of a T-tub to allow caveola identification beyond doubt (as shown in Fig. 5) or perpendicularly through a clearly identifiable T-tub (as, for example, in Fig. 5 *C* of (65)), the fraction of identifiable T-tub caveolae is further reduced by about one order of magnitude, i.e., well below 1%.

**Figure 8:**
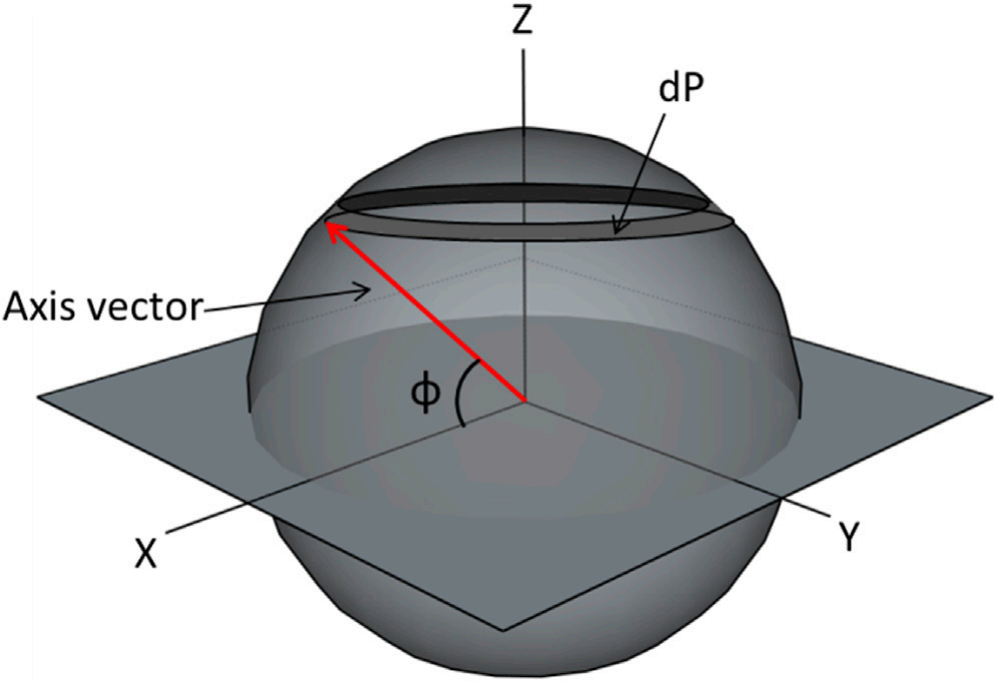
Shown is the elemental area, *dP*, which is directly proportional to the probability of the cylinder axis having orientation angle *φ*.

**Figure 9:**
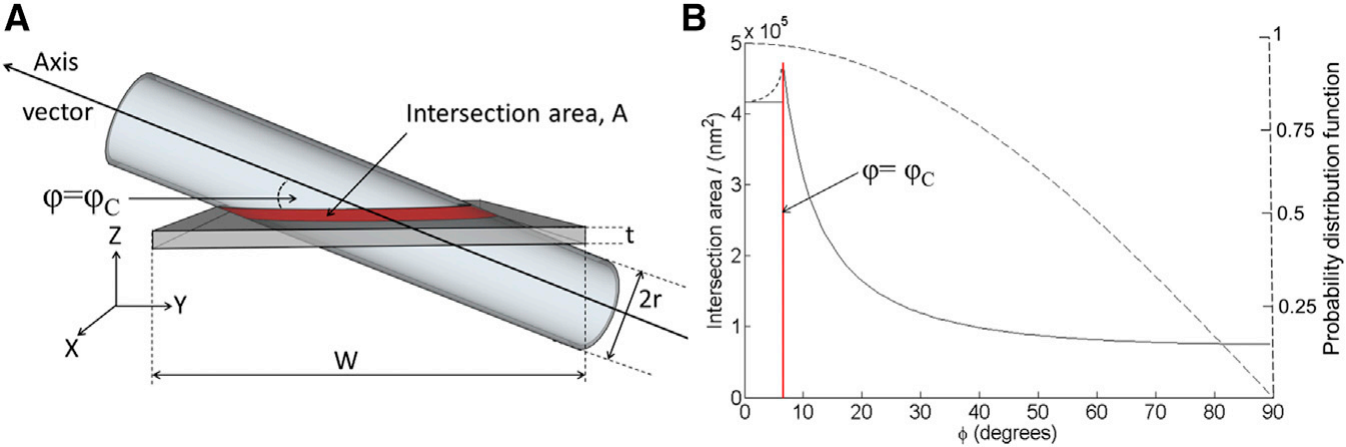
(*A*) Model of the intersection area, *A*, of a cylindrical structure (T-tub) of radius *r* and a slab (TEM image section) of width *W* and thickness *t*. The cylinder axis makes an angle *φ* with the plane of the slab and the cylinder is shown oriented at the critical angle (*φ* = *φ*_C_). (*B*) The solid line describes the variation in the interface area, *A* (*left axis*), as a function of the inclination angle of the cylinder, *φ*, as predicted by the model. The small-dashed black line predicts the variation in intersection area for *φ* < *φ*_C_. The large-dashed line indicates the probability distribution function (*right axis*) for the cylinder orientation as a function of *φ*.

Thus, to successfully identify caveolae from a TEM image, caveolae must 1) be present (expectation here: surface-sarcolemmal and T-tub caveolae may be relatively evenly matched per whole-cell section), 2) be cut at the right location (center of neck region, also with the expectation that this may occur equally frequently for surface-sarcolemmal and T-tub caveolae), and 3) be cut at an amenable angle (which is likely to occur with a two to three orders of magnitude higher probability for surface-sarcolemmal caveolae compared to T-tub caveolae). This is before taking into consideration that user-“optimization” of cardiac cell TEM data will also optimize surface-sarcolemmal caveolae detection (given the surface-normal orientation of surface-sarcolemmal caveolae: if you see one, you see all that are contained in a section and its serially cut neighbors, something that cannot be done for T-tub caveolae, even if it was desired). See appendix (Figs. 8 and 9) for more details.

## Discussion

Caveolae are centers of cardiac signal transduction, frequently studied in isolated cells. Although changes in cardiomyocyte structure (such as shape) and ultrastructure (e.g., T-tub integrity (53)) have been reported after cell isolation (though generally thought to become relevant over days, not hours) (28, 66), little is known about caveolae preservation in isolated myocytes. Here, we report significant changes in surface-sarcolemmal caveolae distribution within hours of cell isolation, with reductions in surface-sarcolemmal caveolae numbers by one-third within 3 h, and down to half by 8 h. This marked change has a potentially significant impact on functional studies conducted on isolated cells over matching time periods. Among the types of studies potentially affected by changing caveolae distribution over time are electrophysiological investigations (e.g., via caveolar effects on calcium-induced calcium release (67)), studies of intracellular signaling (e.g., via G-protein coupled receptors (68, 69), adrenergic and nitric-oxide-mediated pathways (70, 71), platelet-derived growth factor and epidermal growth factor receptor (72)), and experimental research into mechano-sensing (23). Additionally, one should keep in mind the potential impact of changes in caveolae distribution on cytoskeletal function (21, 73, 74, 75).

There are several potential functional implications of the result of our study. Caveolae can act as a membrane reserve that buffers membrane tension and protects the membrane from rupture. Loss of 75% of caveolae from the cardiac myocyte membrane by methyl-*β*-cyclodextrin has been shown to enhance mechanotransductive processes and cell lysis in response to cell swelling (76). In addition, the same degree of caveolar disruption enhances the contractile response to *β*2-adrenergic stimulation in cardiac cells, through the loss of normal compartmentalization of signal transduction components concentrated within the caveolar microdomain (77). The profound effect of a 75% reduction of caveolae on cardiac myocyte function suggests that the 50% loss of caveolae we report here will have functional consequences.

Reduction in surface-sarcolemmal caveolae could be occurring by internalization, loss to the extracellular space, or via incorporation of caveolae into the surface sarcolemma. In support of the latter scenario, we observed an increase in cell capacitance in the first 8 h after isolation, suggesting an increase in the total surface membrane area. The increased membrane capacitance and altered membrane surface-to-volume relationship could lead to changes in the charge required to depolarize the membrane, reduced upstroke velocity, and slowing of conduction (29). This should be taken into account in research targeted at, or affected by, caveolae structure and function.

Since cardiomyocytes swell after isolation (78), even in iso-osmotic conditions (attributed predominantly to oncotic pressure gradients), it is possible that associated mechanical factors contribute to the destabilization of caveolar structures, with membrane incorporation (“flattening”) of caveolae, as reported previously in rabbit ventricular tissue subjected to volume overload (stretching cells) or hyposmotic perfusion (swelling cells) (28, 30, 33). This is in keeping with indications of mitochondrial swelling, observed in SEM images of cell surface topology. As sub-sarcolemmal mitochondria are constrained from centripetal movement by the contractile filament lattice (79), their swelling could enhance local stretch of the cell membrane, perhaps aiding incorporation of surface-sarcolemmal caveolae into the sarcolemma.

In addition to surface-sarcolemmal caveolae, we document the regular presence of T-tub caveolae in T-tub membranes. Diameter and volume of T-tub caveolae are indistinguishable from those of surface-sarcolemmal caveolae, though T-tub caveolae have a roughly fourfold lower density per host membrane area (∼3 T-tub caveolae per 1 *μ*m^2^ T-tub membrane area) compared to surface-sarcolemmal caveolae (∼12 per 1 *μ*m^2^ sarcolemma). In either case, membrane contained in caveolae represents an additional 6–24% membrane “reserve” (3–12 × 0.02 *μ*m^2^ per 1 *μ*m^2^ of host membrane), based on our rabbit ventricular ET data.

Caveola-like shapes have been observed in close proximity to T-tubs in earlier TEM studies (37, 38, 80). However, their existence has remained controversial (81). This is in part due to the technical challenges associated with reliable T-tub caveolae identification in typical 80 nm thin sections, as no prior studies have assessed T-tub caveolae in 3D EM tomography data. As an illustration, Fig. 6 documents what one bona fide T-tub caveola, identified by ET, would look like in a series of 2D TEM sections at different imaging planes. This highlights that without 3D tracing it is difficult to identify the presence of T-tub caveolae and their interrelation with the T-tub host membrane.

Early studies by Parton et al. (40) found that Cav-3 is transiently associated with T-tubs during development. It has been suggested that caveolae are required for the formation of the T-tub system via repetitive generation of inwardly directed sarcolemmal membrane nano-bulges (the “beaded tubules” theory) (40, 82, 83, 84). Supporting this theory is the fact that both T-tubs and caveolae share similarities in protein and lipid composition that are considerably different from those of the surface sarcolemma (41). Furthermore, Cav-3 null mice show not only loss of caveolae, but also abnormalities in T-tub system organization (in skeletal muscle), such as dilatation and loss of transverse orientation (12), and Cav-1/Cav-3 double-knockout mice show complete loss of cardiac caveolae, T-tub disorganization, and severe cardiomyopathy (12, 14). In contrast, in adult myocytes, it has been proposed that T-tub Cav-3 forms scaffolds rather than morphologically identifiable caveolar structures (70). Since T-tubs are an important determinant of cardiac cell function, and changes in their structure and protein expression occur during development and heart failure (see review in (85)), the presence of caveolae (and their associated proteins) on the T-tub could potentially contribute to important physiological and pathological functions. Whether T-tub caveolae serve as signaling centers, and to what extent this may involve mechano-sensitive alterations in T-tub structure during the cardiac cycle (similarly to CAVs (27, 28, 29)), remains to be elucidated; the technology to do so exists (86, 87).

EM tomography has been used in this study because it is the only imaging method capable of resolving caveolar arrangements in 3D, which is necessary to discover and quantify these very small structures. Data from EM tomography compared favorably with those of more conventional techniques, such as TEM and SEM. To conclude, we 1) report dynamic remodeling of surface-sarcolemmal caveolar structures in rabbit ventricular cardiomyocytes post-isolation and provide functional assessment indicative of electrophysiological consequences, 2) establish that this occurs over a 3- to 8-h time course, which is relevant for research using acutely isolated cells, 3) confirm the additional regular presence of caveolae on membranes of the T-tub system, and 4) suggest an explanation for why the latter may have been overlooked in previous ultrastructural research. It will be important now to investigate potential strategies to preserve caveolar integrity in isolated cells. Given the huge parameter space for possible modifications, a useful step would come from raising awareness of this as a variable parameter that should be assessed when evaluating existing cell isolation protocols by the different teams using acutely isolated cardiac cells.

## Author Contributions

R.A.B.B., E.A.R.-Z., G.B., and P.K. designed research. R.A.B.B., E.A.R.-Z., R.P., I.B., M.F., and J.S. performed research. A.D.C., A.H., and G.B. contributed analytic tools. R.A.B.B., E.A.R.-Z., R.P., I.B., A.D.C., and S.C.C. analyzed and interpreted data. R.A.B.B., E.A.R.-Z., A.D.C., S.C.C., and P.K. wrote the article; and all authors read and approved the submitted manuscript.

## Appendix A: Formulating A Description of the Intersection Area, A

Starting with the axis of the cylinder oriented normally to the image plane (*φ* = 90°) and then gradually reducing the value of *φ*, it can be seen that a point is reached at which the full width of the image section is completely contained within the cylinder. At this point, the diagonal of the image section makes an angle, *φ*_C_ (the critical angle), with the axis of the cylinder. The variation in *A* with the rotation angle *φ* can then be conveniently broken down into two regimes, first for *φ* > *φ*_C_ and then for *φ* ≤ *φ*_C_. For *φ* > *φ*_C_, the intersection area is labeled *A*_1_ and for *φ* ≤ *φ*_C_, the intersection area is labeled *A*_2_.

For *φ* > *φ*_C_, the image section always extends beyond the length of the cylinder, with the intersection area reaching a minimum at *φ* = 90°. For values <90°, the perimeter of the intersection area will describe an ellipse, with minor axis *a* = *r* and major axis *b* = (*r*/sin(*φ*)). A first approximation to *A* will therefore be given by the product of the circumference of the ellipse, *C*, and the sectioning thickness, *t*. The parameter *C* can be approximated using the expression given in Eq. 1:

(1) $$C\approx 2\pi \times {\left[0.5\times \left(a^{2}+b^{2}\right)\right]}^{\frac{1}{2}}.$$

Making substitutions for *a* and *b*, we therefore have

(2) $$A_{1}=C\times t=2\pi \times r\times t\times {\left[0.5\times \left(1+{\left(1/\text{sin}\left(\phi \right)\right)}^{2}\right)\right]}^{\frac{1}{2}}.$$

It can be seen that for *φ* = 90°, *A* = 2*πrt*, which is as expected from the product of the cylinder circumference (2*πr*) and the section thickness, *t*. The expression in Eq. 2 describes the regime for *φ* > *φ*_C_. For *φ* ≤ *φ*_C_, the intersection area is maximized, as the image section is completely contained within the walls of the cylinder. Introducing a factor of 2 to account for the intersection areas on both sides of the cylinder, we can approximate the intersection area as

(3) $$A_{2}=2\times W\times t.$$

### Calculating the expectation value of A

As not all orientations of the cylinder are equally likely, we need to modulate the above expressions for *A* by the probability, *dP*, of the cylinder having angle *φ*, to form the expectation value for *A*. The first task is then to form a probability distribution function that describes the likelihood of a randomly oriented cylinder being inclined at angle *φ* with respect to the image plane. For a purely random orientation, the endpoint of a vector running parallel to the cylinder axis (the axis vector) will describe a spherical shell of uniformly distributed points (Fig. 8).

The probability of a given angle, *φ*, will therefore be proportional to the area, *dP*, on the sphere which is given by

(4) dP=2π×cos(ϕ)×dϕ.

We now have sufficient information to calculate the expectation value for the intersection area:

(5) $$\langle A\rangle =\langle A_{1}\rangle +\langle A_{2}\rangle =\frac{2}{4\pi }\times \left[\int_{\varphi_{\text{C}}}^{\pi /2}A_{1}\times dP+\int_{0}^{\varphi_{\text{C}}}A_{2}\times dP\right].$$

In Eq. 5, we have normalized by 4*π* to ensure that the total probability sums to unity. The first expression for *A*_1_ can be written as

(6) $$\langle A_{1}\rangle =\frac{2}{4\pi }\times \left[\int_{\phi_{\text{C}}}^{\pi /2}2\pi rt{\left[0.5\times \left(1+{\left(1/\text{sin}\left(\phi \right)\right)}^{2}\right)\right]}^{\frac{1}{2}}\times \text{cos}\left(\phi \right)d\phi \right].$$

It is possible to simplify this expression by setting *x* = sin(*φ*):

(7) $$\langle A_{1}\rangle =\frac{rt}{\sqrt{2}}\times \int_{\text{sin}\left(\phi_{\text{C}}\right)}^{1}{\left(1+{\left(1/x\right)}^{2}\right)}^{\frac{1}{2}}\times dx.$$

Equation 7 describes an elliptical integral that can be represented as

(8) $$\langle A_{1}\rangle =\frac{2\pi \times rt}{\sqrt{2}}\times {\left[\frac{x\sqrt{1+{\left(1/x\right)}^{2}}}{\sqrt{x^{2}+1}}\left(\sqrt{x^{2}+1}-\text{log}\left(1+\sqrt{x^{2}+1}\right)+\text{log}\left(x\right)\right)\right]}_{\text{sin}\left(\phi_{\text{C}}\right)}^{1}.$$

Evaluating this function for the experimental value of *φ* = 6.6°, we get

(9) $$\langle A_{1}\rangle =10.6\times rt=1.28\times 10^{5}\;{\text{nm}}^{2}.$$

We can now calculate the expectation value of *A*_2_, which is much more straightforward:

(10) $$\langle A_{2}\rangle =\int_{0}^{\phi_{\text{C}}}2Wt\times \text{cos}\left(\phi \right)d\phi =2Wt\times \text{sin}\left(\phi_{\text{C}}\right)=4.8\times 10^{4}\;{\text{nm}}^{2}.$$

Combining the results from Eqs. 9 and 10 we have a total expectation value of

(11) $$\langle A\rangle =1.75\times 10^{5}\;{\text{nm}}^{2}.$$

### Modeling the likelihood of detecting T-tub caveolae

Using a simplistic model, one can estimate the probability of detecting a caveola by pure chance in a cylindrical structure (e.g., a T-tub), which has a radius, *r,* and a length that extends beyond the field of view of the TEM image. In this model, it is assumed that T-tub caveolae (abbreviated CAV_T_ in equations) will be imaged if they lie within an area described by the intersection of the cylinder and a rectangular slab of width *w* and thickness *t*, corresponding to the TEM image section (see Fig. 9 *A*). Assuming that T-tub caveolae can be randomly distributed across the surface area of the cylindrical structures, the likelihood of seeing the caveolae in close proximity to the T-tub varies in direct proportion with the size of the intersection area, *A*. This model determines how *A* varies with the relative orientation of the cylindrical structure and the image section. In principle, the orientation of either the cylinder or the image section can be changed. In this description, it is the cylinder that is reoriented, forming an angle *φ* between the cylinder axis and the plane of the image section (Fig. 9 *A*).

A plot of *A*(*φ*) can be seen in Fig. 9 *B*. Here, we have substituted experimental values for *t*, *r*, and *W* (80, 150, and 2600 nm, respectively). It can be seen that the model of *A*(*φ*) has been split into two parts, *A*_1_ and *A*_2_, depending upon whether the inclination angle of the cylinder is above or below the critical angle (demonstrated in Fig. 9 *A*) at which *φ* = sin^−1^(2*r*/*W*). The mismatch between the models for *A*_1_ and *A*_2_ at *φ* = *φ*_C_ is a consequence of the simplicity of the model for *A*_2_, which underestimates *A* for *φ* > 0. A more sophisticated model would appear like the small-dashed line in Fig. 9 *B*, although, as is clear from the figure, this does not significantly change the result.

The function describing *A*(*φ*) can be multiplied by a probability distribution, which determines the likelihood of a randomly oriented cylinder having an orientation of *φ* (Fig. 9 *B*, *large-dashed line*). Integrating this product over all possible values of *φ* delivers the expectation value of *A*, which is to say the statistical average value of the intersection area. For the parameter values applicable to the above experiment, the expectation value for a single T-tub was calculated to be (see Appendix for full details)

(12) $$\langle A_{\text{T}}\rangle =0.175\;\mu {\text{m}}^{2}.$$

From the measured surface density value of 3.23 T-tub caveolae per *μ*m^2^ of T-tub outer surface it is possible to calculate the expectation value for the number of T-tub caveolae seen in an average TEM image of a randomly oriented T-tub:

(13) $$\langle {\text{CAV}}_{\text{T}}\rangle =0.175\;\mu {\text{m}}^{2}\times 3.23\;{\text{CAV}}_{\text{T}}/\mu {\text{m}}^{2}=0.57\;{\text{CAV}}_{\text{T}}.$$

This tells us that for a randomly oriented T-tub, we would on average expect to image around one T-tub caveola every two TEM images. This calculation assumes only a single T-tub within the 2.6 × 2.6 *μ*m field of view of each TEM image, which is likely to be different in most cases, depending on the deviation of the cutting plane from a cut perpendicular to T-tub orientation. Nonetheless, this simplified analysis implies that T-tub caveolae should be a common feature in TEM images. This issue is further explored in the section Presence of Surface-Sarcolemmal Caveolae and T-Tub Caveolae in TEM Images: Spatial Density Considerations.

## Supporting Material

Document S1. Fig. S1

Movie S1. Surface Membrane Caveolae Modelling

Movie S2. T-Tub Caveolae Modelling

Document S2. Article plus Supporting Material
