# Supplementary material for: Caveolae in Rabbit Ventricular Myocytes: Distribution and Dynamic Diminution after Cell Isolation
Source: Biophys J. 2017 Sep 5;113(5):1047–59. doi: 10.1016/j.bpj.2017.07.026 (PMC5653872; doi:10.1016/j.bpj.2017.07.026)

Biophysical Journal, Volume *113*

Supplemental Information

Caveolae in Rabbit Ventricular Myocytes: Distribution and Dynamic Diminution after Cell Isolation

Rebecca A.B. Burton, Eva A. Rog-Zielinska, Alexander D. Corbett, Rémi Peyronnet, Ilona Bodi, Martin Fink, Judith Sheldon, Andreas Hoenger, Sarah C. Calaghan, Gil Bub, and Peter Kohl

Supplementary Material

Supplementary Figure S1. Whole cell Cav-3 protein levels are reduced after 8h, as shown by Western blot of isolated cell extracts (left, n = 3 cell isolations; data analysed using ANOVA, significance indicated for the overall effect of time) and measurements of fluorescence intensity in isolated cells (right, n = 18 cells from 3 isolations for each time point; data analysed using ANCOVA, # p=0.002 vs 0h; scale bar = 20 µm).

Whether this is indicative of internalisation and protein degradation, or loss to the extracellular space, requires further elucidation.

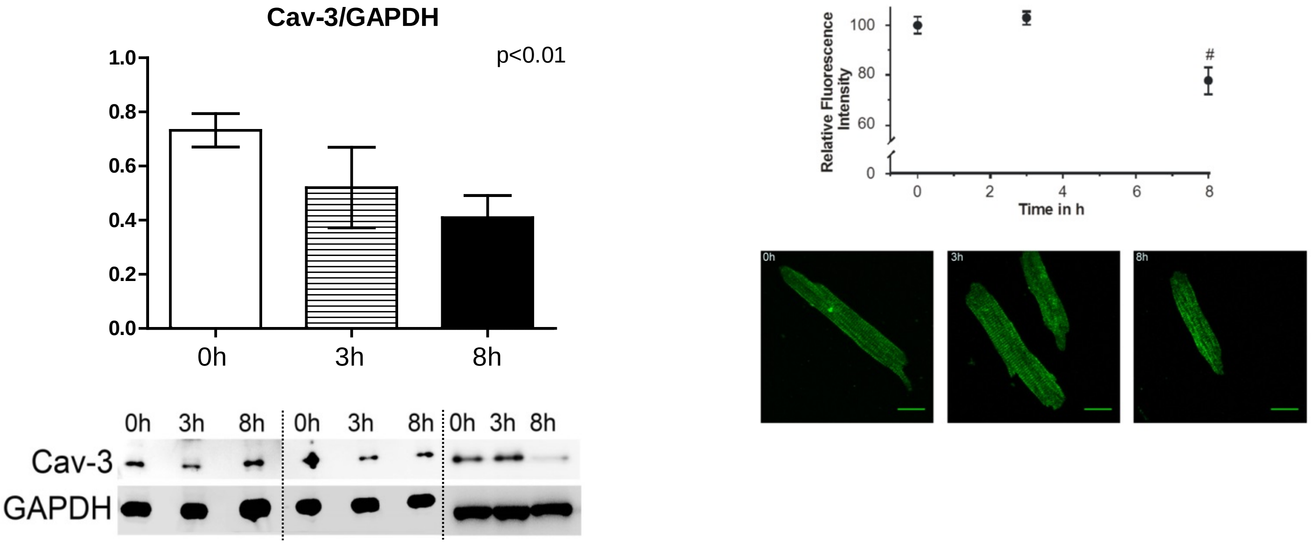

Supplement: Document S1. Fig. S1 [file mmc1.pdf]
